# ADAM10 mediates malignant pleural mesothelioma invasiveness

**DOI:** 10.1038/s41388-018-0669-2

**Published:** 2019-01-16

**Authors:** Christelle Sépult, Marine Bellefroid, Natacha Rocks, Kim Donati, Catherine Gérard, Christine Gilles, Andreas Ludwig, Bernard Duysinx, Agnès Noël, Didier Cataldo

**Affiliations:** 1 Laboratory of Tumour and Development Biology, GIGA-cancer, Liège University, Avenue Hippocrate 13, 4000 Liège, Belgium; 2 Institute of Pharmacology and Toxicology, RWTH Aachen University, Aachen, Germany; 3 Department of Respiratory diseases, CHU Liège and Liège University, Avenue Hippocrate 13, 4000 Liège, Belgium

**Keywords:** Mesothelioma, Targeted therapies, Cell migration, Mechanisms of disease

## Abstract

Malignant pleural mesothelioma (MPM) is an aggressive cancer with limited therapeutic options and treatment efficiency. Even if the latency period between asbestos exposure, the main risk factor, and mesothelioma development is very long, the local invasion of mesothelioma is very rapid leading to a mean survival of one year after diagnosis. ADAM10 (A Disintegrin And Metalloprotease) sheddase targets membrane-bound substrates and its overexpression is associated with progression in several cancers. However, nothing is known about ADAM10 implication in MPM. In this study, we demonstrated higher ADAM10 expression levels in human MPM as compared to control pleural samples and in human MPM cell line. This ADAM10 overexpression was also observed in murine MPM samples. Two mouse mesothelioma cell lines were used in this study including one primary cell line obtained by repeated asbestos fibre injections. We show, in vitro, that ADAM10 targeting through shRNA and pharmacological (GI254023X) approaches reduced drastically mesothelioma cell migration and invasion, as well as for human mesothelioma cells treated with siRNA targeting ADAM10. Moreover, ADAM10 downregulation in murine mesothelioma cells significantly impairs MPM progression in vivo after intrapleural cell injection. We also demonstrate that ADAM10 sheddase downregulation decreases the production of a soluble N-cadherin fragment through membrane N-cadherin, which stimulated mesothelioma cell migration. Taken together, we demonstrate that ADAM10 is overexpressed in MPM and takes part to MPM progression through the generation of N-cadherin fragment that stimulates mesothelioma cell migration. ADAM10 inhibition is worth considering as a therapeutic perspective in mesothelioma context.

## Introduction

Malignant mesothelioma is a rapidly growing invasive cancer derived from mesothelial cells that mainly affects pleural tissues [1]. Airborne asbestos exposure is the primary cause for pleural mesothelioma and crocidolite is known as the most oncogenic type among asbestos fibres [2]. Although the latency between asbestos exposure and malignant pleural mesothelioma (MPM) development lasts about 30 to 40 years, tumour progression is very rapid once MPM is established, leading to an important local invasion followed by a thickening of all pleural surfaces [3]. The MPM patient prognosis is very poor and available therapies have still a limited impact on MPM progression [4]. Hence, a better understanding of molecular mechanisms supporting MPM cell proliferation, invasiveness and dissemination is urgently needed to decipher disease mechanisms and design new therapeutic targets against this fatal disease.

A Disintegrin And Metalloprotease (ADAMs) constitute a group of transmembrane proteases belonging to the superfamily of metzincins [5]. Among these proteases, ADAM10, whose overexpression has been reported in several cancers [6–10], displays numerous features that can be helpful in advancing cancer research. ADAM10 has been described to impact tumour progression, chemoresistance, metastasis and invasion [11–15]. One of the key features of ADAM10 is its sheddase activity that can generate ectodomain cleavage of various surface molecules such as growth factors, receptors and adhesion proteins, all reported to display crucial roles in tumour progression [13–15]. ADAM10 cleaves adhesion proteins such as CD44, E-cadherin, N-cadherin and L1 adhesion molecules, whose functions have been correlated with tumour cell migration and invasion [15]. N-cadherin is a type I transmembrane glycoprotein implicated in cell-cell interactions that regulates the migratory behaviour of healthy and cancer cells [16]. N-cadherin cleavage by ADAM10 generates a soluble N-cadherin fragment which has been depicted to promote angiogenesis in cornea and chorioallantoic assays [17], neurite growth [18, 19], cell motility of squamous epithelial cells [20] and glioblastoma cell migration [21]. All these features of ADAM10 support our hypothesis of a role for ADAM10 in progression of MPM.

In this study, we provide for the first time evidence that ADAM10 is overexpressed in human MPM samples and, by using experimental mouse models of mesothelioma development, we demonstrate the link between ADAM10 and MPM progression. In vitro and in vivo experiments using ADAM10 shRNA, siRNA or an ADAM10 pharmacological inhibitor support the importance of ADAM10 in MPM development. In addition, we report that the inhibition of the ADAM10-dependant generation of a soluble N-cadherin ectodomain fragment leads to reduced mesothelioma cell migration. Taken together, our results indicate that ADAM10 is implicated in MPM progression and mediates tumour cell invasiveness via N-cadherin cleavage.

## Results

### ADAM10 is overexpressed in human MPM

**Fig. 1:**
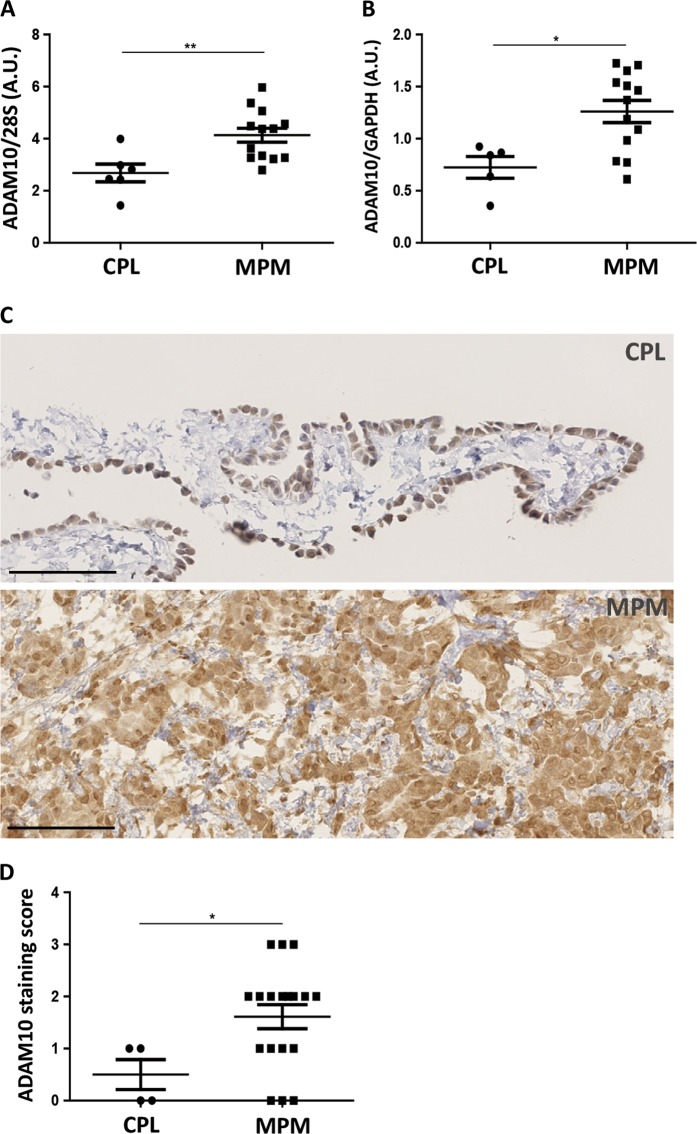
ADAM10 expression levels in human samples of control pleura (CPL) and malignant pleural mesothelioma (MPM). **a** RT-PCR measurement of ADAM10 mRNA levels in human MPM (*n* = 13) and CPL (*n* = 6) samples. Quantification of ADAM10 mRNA expression was normalized to 28S rRNA. Results are expressed as mean ± SEM. Statistical significance was calculated by Student *t* test; **P = 0.0056. **b** Measurement of ADAM10 protein levels by western blotting in human samples of CPL (*n* = 5) and MPM (*n* = 13). Quantification of ADAM10 protein expression was normalized to GAPDH protein. Results are expressed as mean ± SEM. Statistical significance was calculated by Student *t* test; *P = 0.0104. **c** Representative examples of immunohistochemistry targeting ADAM10 in CPL and in MPM tissues; scale bars = 100 µm. **d** Score of staining intensity (from 0 to 3) of immunohistochemistry targeting ADAM10 on CPL (*n* = 4) and MPM (*n* = 18) tissues. Results are expressed as mean ± SEM. Statistical significance was calculated by Student *t* test; *P = 0.0429

To analyse the expression of ADAM10 in MPM, human samples of MPM and control pleura (CPL) were collected (Table 1). A stronger expression of ADAM10 was detected in human MPM as compared to CPL samples at mRNA (Fig. 1a) and protein levels (Fig. 1b). These data were corroborated with immunohistochemical analysis targeting ADAM10, which showed a stronger staining in MPM tissues as compared to normal mesothelium (Fig. 1c, d).

**Table 1:** Patient sample characteristics

	Frozen samples		Paraffin-embedded samples	
	CPL (*n* = 6)	MPM (*n* = 13)	CPL (*n* = 4)	MPM (*n* = 18)
Age (years)				
Mean ± SD	37 ± 18.8	72.8 ± 8.4	42.7 ± 21.2	72.6 ± 11.5
Sex (%)				
Male	5 [83]	11 [84,6]	3 [75]	14 [77,8]
Female	1 [17]	2 [15,4]	1 [25]	4 [22,2]
Histotype MPM (%)				
Epithelioid		7 [53,8]		13 [72,2]
Biphasic		4 [30,8]		3 [16,7]
Sarcomatoid		2 [15,4]		2 [11,1]

*CPL* control pleura, *MPM* malignant pleural mesothelioma

### ADAM10 is overexpressed in mouse mesothelioma cells and in pleural mesothelioma in vivo

**Fig. 2:**
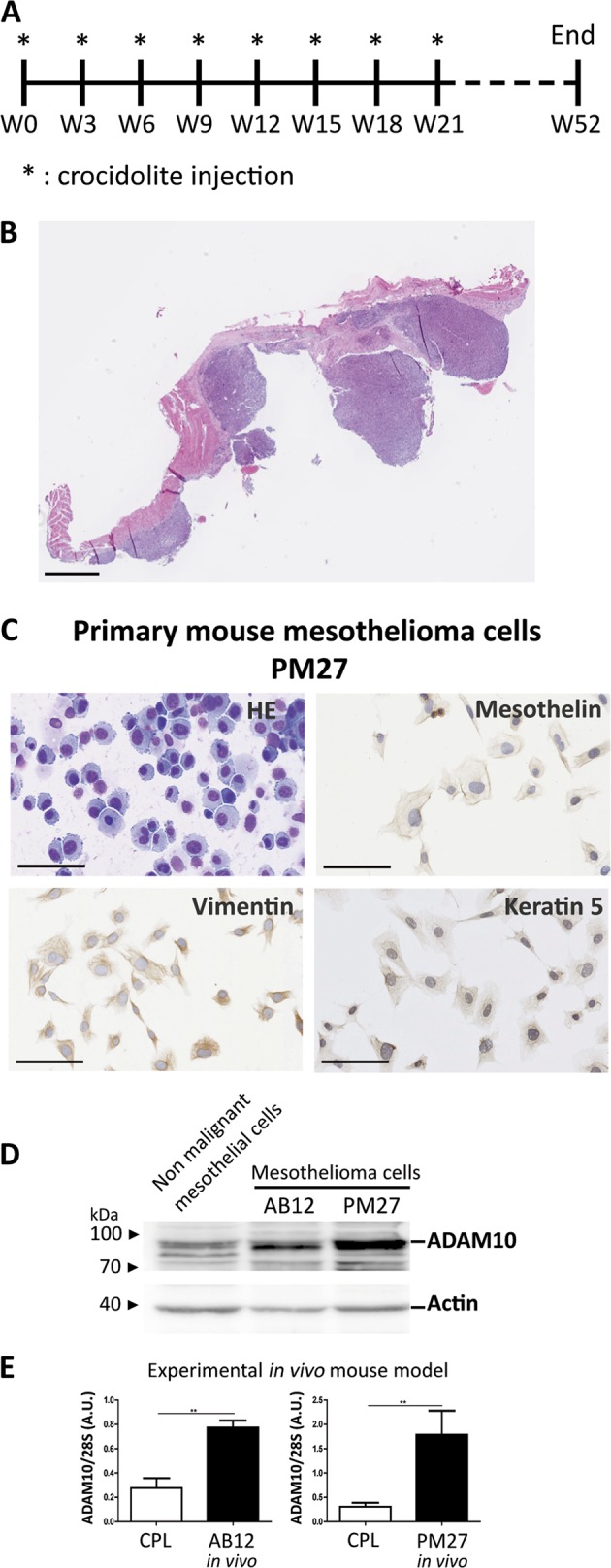
Development of a primary mesothelioma (PM) cell line and expression levels of ADAM10 in mouse mesothelioma. **a** Timeline of the experimental mouse model of primary mesothelioma development. Mice were intraperitoneally injected with crocidolite fibres every three weeks (stars). A total of 8 injections were performed. Primary mesothelioma occurred maximum one year after the first crocidolite injection. **b** Hematoxylin-eosin (HE) staining of primary mesothelioma nodules developed on diaphragm of crocidolite-treated mice; scale bar = 1 mm. **c** Representative images of primary mesothelioma PM27 cell line characterization: HE staining and immunocytochemistry targeting mesothelin, vimentin and keratin 5; scale bars = 100 µm. **d** Measurement of ADAM10 expression by western blotting in murine non-malignant mesothelial cells and in mesothelioma cell lines (AB12 and PM27). Actin was used as loading control. **e** Quantification of ADAM10 mRNA expression by RT-PCR in control pleura (CPL, *n* ≥ 3) from diaphragm of healthy mice and in mouse mesothelioma tumours obtained after intrapleural injection of AB12 (*n* = 6) or PM27 (*n* = 5) cells. ADAM10 expression levels were normalized to 28S rRNA levels. Results are expressed as mean ± SEM; Student *t* test; **P < 0.01

In order to expand our mesothelioma tools, we aimed at establishing primary mesothelioma cells (PM) from ascites developed in mice after repeated intraperitoneal injections of crocidolite fibres (one injection every three weeks, eight in total) leading to the development of mesothelioma (Fig. 2a, b). PM cells, referred as PM27 in the present work, express mesothelin, vimentin and keratin 5, considered as typical markers of mesothelioma (Fig. 2c) [22]. As control, primary non-malignant mouse mesothelial cells were isolated from naive peritoneum. These control mesothelial cells survived only three passages while PM27 cells could be maintained as a cell line, attesting of their immortalization. Analysis of ADAM10 expression in mesothelioma PM27 cells and in commercial mesothelioma AB12 cells revealed increased ADAM10 protein production as compared to non-malignant mesothelial cells (Fig. 2d). Strikingly, ADAM10 remained overexpressed in mesothelioma tumours induced by the in vivo injection of AB12 or PM27 cells as compared to CPL isolated from diaphragms of healthy mice (Fig. 2e).

### ADAM10 depletion by shRNA decreases mesothelioma cell migration and invasion

**Fig. 3:**
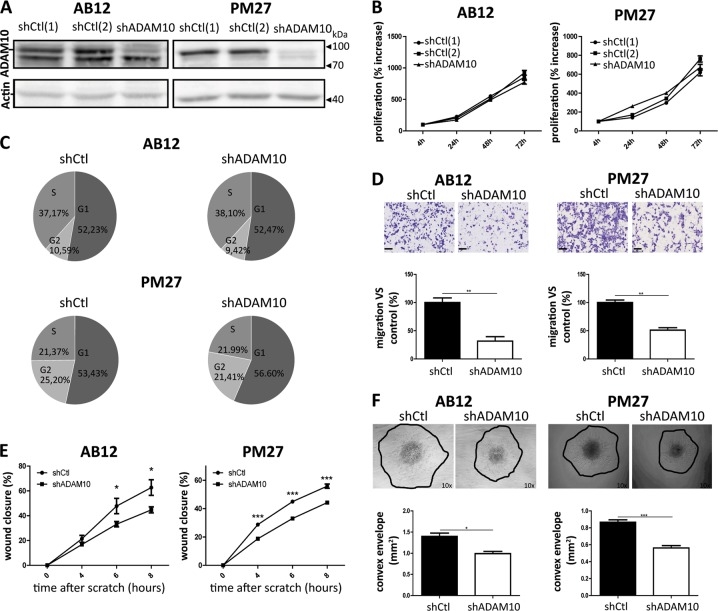
Effects of ADAM10 shRNA depletion in mesothelioma cells on proliferation, migration and invasion. **a** Western blot analysis of ADAM10 protein levels in AB12 and PM27 cells transduced with two different control shRNA (shCtl[1] or [2]) or with a shRNA targeting ADAM10 (shADAM10). Actin was used as a loading control. **b** Proliferation rates were assessed for AB12 and PM27 cells after 4, 24, 48 and 72 h by CYQUANT analysis (*n* ≥ 6). All measurements were normalized to the ‘4 h-timing’ considered as baseline proliferation and expressed as percentage increase from baseline. For in vitro results, shCtl condition correspond to shCtl[1]-treated AB12 or PM27 cells. **c** Representative examples of AB12 and PM27 percentages present in the different phases of cell cycle (G1, S or G2) 24 h after cell seeding. **d** Upper panel: representative HE-stained images showing cells that migrated through the transwell filter. Scale bars = 100 µm. Lower panel: quantification of numbers of migrating AB12 or PM27 cells transduced with shCtl or shADAM10 per field (*n* = 3). Results are expressed as percentage of control condition (mean ± SEM); Student *t* test; **P < 0.01. **e** Wound healing assay performed on AB12 and PM27 cells transduced with shCtl or shADAM10. The wound closure was evaluated 4, 6 and 8 h after the scratch (*n* = 8). Results are expressed as mean ± SEM; Student *t* test; *P < 0.05; ***P < 0.001. **f** Upper panel: representative examples of spheroids composed of AB12 or PM27 cells transduced with shCtl or shADAM10; magnification ×10. Lower panel: quantification of cell invasion in collagen gel (*n* ≥ 7). Results are expressed as mean ± SEM; Student *t* test; *P < 0.05; ***P < 0.001

In order to examine the potential implication of ADAM10 in mesothelioma tumour progression, AB12 and PM27 were transduced with an ADAM10 shRNA lentiviral vector, which, as expected, strongly decreased ADAM10 production (Fig. 3a). To characterize the phenotype of shADAM10-transduced cells, cell proliferation rates, migration or invasion patterns were analysed. ADAM10-downregulated AB12 and PM27 cells displayed similar proliferation rates than shCtl-treated control cells (Fig. 3b). For in vitro results, shCtl condition correspond to shCtl[1]-treated AB12 or PM27 cells. Cell cycle distribution (proportion of cells in G1, S and G2 phases) was also similar between experimental groups (Fig. 3c; 24 h after cell seeding). In sharp contrast, mesothelioma cell migration measured in transwell chambers was significantly decreased when AB12 or PM27 cells were transduced with shADAM10 (Fig. 3d). The impact of ADAM10 downregulation on cell migration was further supported by a scratch assay, in which wound closure of shADAM10-treated cells was significantly delayed (Fig. 3e). In addition, invasion capacities of mesothelioma AB12 and PM27 cells from spheroids in a 3D-collagen gel were significantly decreased when ADAM10 expression was inhibited (Fig. 3f).

### ADAM10 depletion by shRNA in mesothelioma cells decreases tumour progression in vivo

**Fig. 4:**
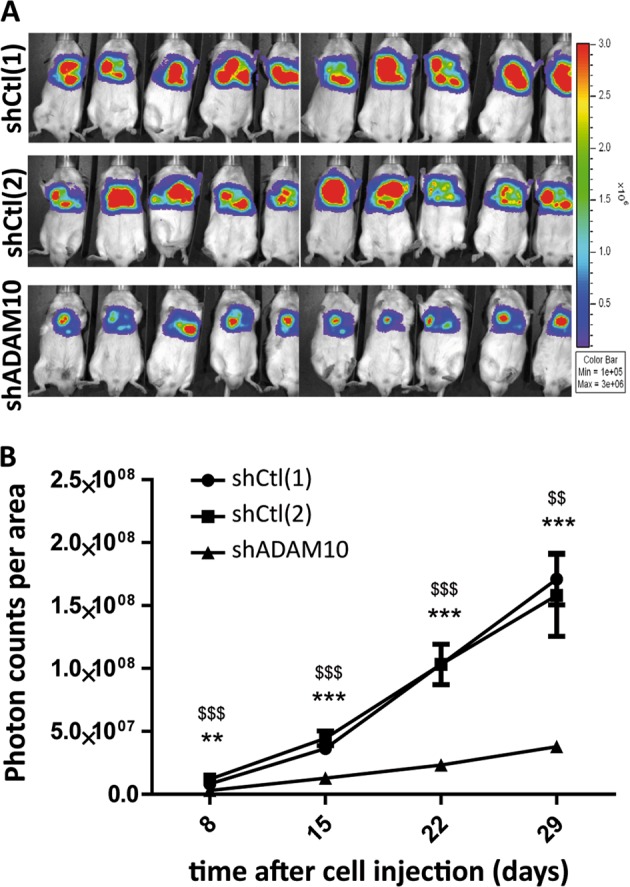
ADAM10-depleted mesothelioma cells decrease in vivo tumour development. **a** Representative bioluminescent images of mesothelioma tumours developed in vivo from AB12 cells depleted (shADAM10) or not (shCtl[1] and [2]) for ADAM10, 29 days after tumour cell injections. **b** Bioluminescence quantification (photon counts per area) of regions of interest (mesothelioma tumours) 8, 15, 22 and 29 days after shCtl[1]-, shCtl[2]- or shADAM10-treated AB12 cell injections (*n* = 10). Results are expressed as mean ± SEM; one-way ANOVA; **P < 0.01, ***P < 0.001 shADAM10 vs shCtl[1]; ^$$^*P* < 0.01, ^$$$^*P* < 0.001 shADAM10 vs shCtl[2]

To assess the impact of ADAM10 depletion in mesothelioma cells in vivo, mice were injected in the pleural cavity with luciferase-expressing AB12 cells, treated with shADAM10 or shCtls. Interestingly, ADAM10 depletion was associated with decreased tumour progression as shown by biophotonic imaging measurements (Fig. 4a, b).

### Pharmacological inhibition of ADAM10 reduces cell migration and invasion

**Fig. 5:**
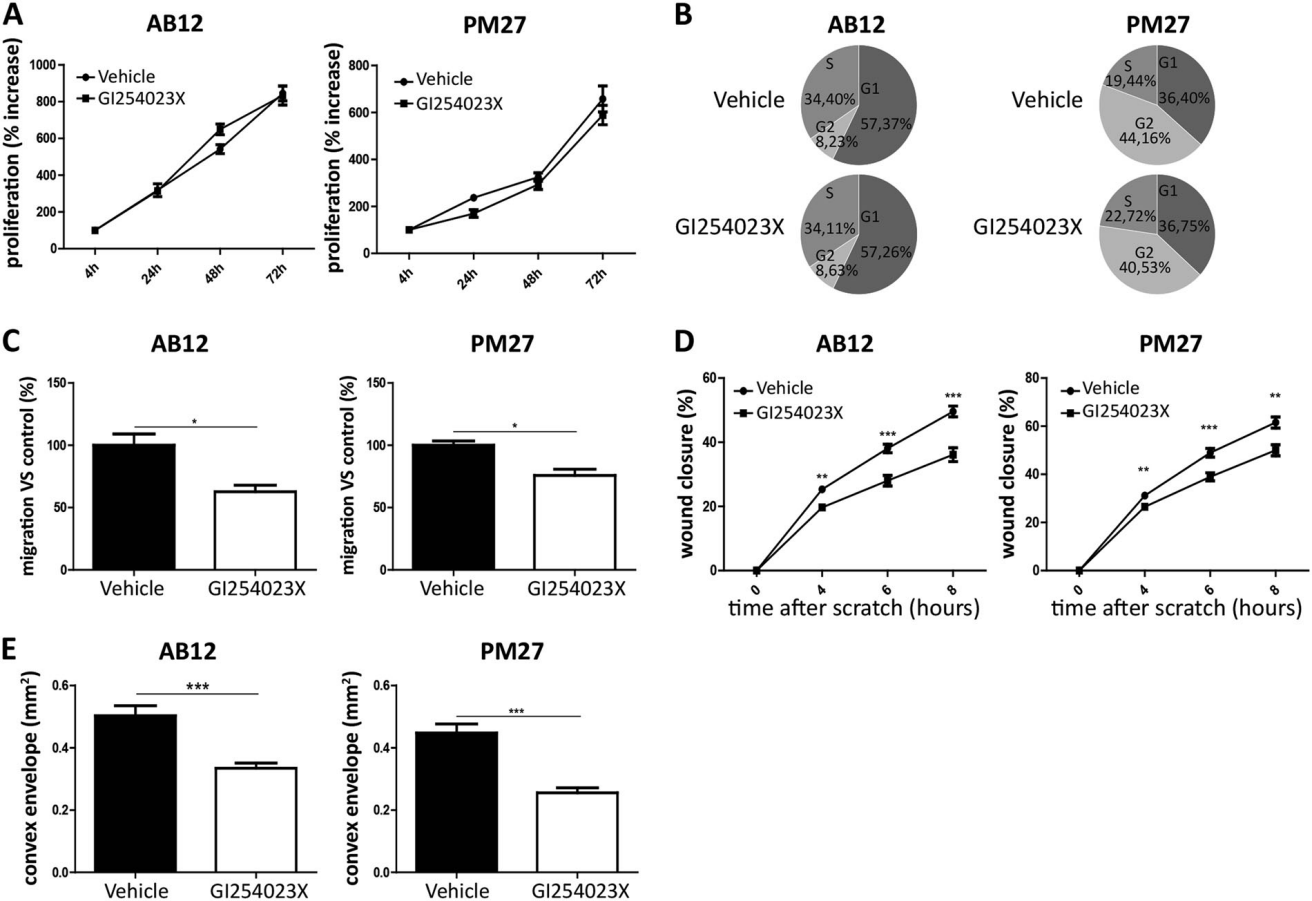
Effects of a pharmacological inhibitor of ADAM10, GI254023X, on proliferation, migration and invasion of mesothelioma cells. **a** Measurement of AB12 (left panel) and PM27 (right panel) cell proliferation by CYQUANT analysis performed 4, 24, 48 and 72 h after treatment with GI254023X (5 µM) or vehicle (*n* = 6). All readings were normalized to the reading ‘after 4 h of treatment’ considered as baseline proliferation and expressed as percentage increase from baseline. **b** Representative examples of percentages of AB12 or PM27 cells in the different phases of cell cycle (G1, S or G2) measured by FACS analysis 24 h after GI254023X (5 µM) or vehicle treatment. **c** Quantification of numbers of migrating AB12 or PM27 cells per field, in response to 5 µM GI254023X or vehicle (*n* = 3). Results are expressed as percentage of control condition (mean ± SEM); Student *t* test; *P < 0.05. **d** Wound healing assay performed on AB12 or PM27 cells treated with GI254023X (5 µM) or vehicle (*n* ≥ 8). The wound closure was evaluated 4, 6 and 8 h after the scratch. Results are expressed as mean ± SEM; Student *t* test; **P < 0.01; ***P < 0.001. **e** Invasive properties of AB12 or PM27 cells treated or not with GI254023X (5 µM) was evaluated by performing a 3D spheroid assay (*n* ≥ 10). Results are expressed as mean ± SEM; Student *t* test; *** *P* < 0.001

To further validate the impact of ADAM10 on mesothelioma cell properties, AB12 as well as PM27 cells were treated with a pharmacological selective ADAM10 inhibitor, GI254023X [6,23], and their phenotypes were analysed accordingly. While treating both cell lines with GI254023X did not modulate proliferation rates (4, 24, 48 and 72 h) (Fig. 5a) or cell cycle distribution (after 24 h of treatment) (Fig. 5b), GI254023X-treated mouse mesothelioma cells displayed weaker migratory properties in transwell chambers as compared to vehicle-treated cells (Fig. 5c), supporting our results obtained with cells expressing ADAM10 shRNA. Similarly, GI254023X-treated mouse mesothelioma cells showed a significantly decreased migration in a scratch assay (at 4, 6 and 8 h) (Fig. 5d). Invasion of AB12 and PM27 cells measured in spheroid assay was also significantly decreased after GI254023X treatment (Fig. 5e).

### ADAM10 depletion impacts N-cadherin cleavage in stimulated mesothelioma cells and further mesothelioma cell migration

**Fig. 6:**
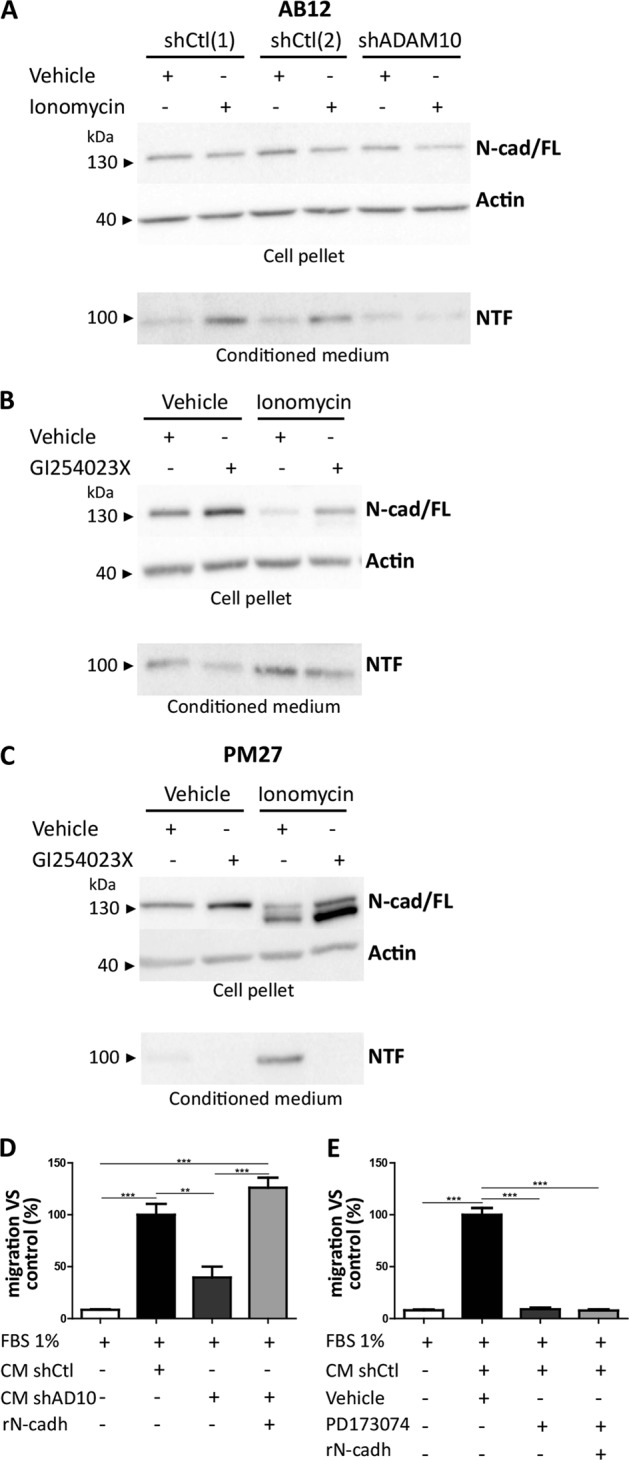
ADAM10 processes N-cadherin shedding in stimulated mesothelioma cells and soluble N-terminal fragment of N-cadherin promotes migration of AB12 mesothelioma cells. **a** Representative gel lanes of western blot analysis of full-length 135 kDa N-cadherin (N-cad/FL) in cell pellets and soluble N-terminal 95 kDa fragment (NTF) in conditioned medium (CM) of stimulated AB12 cells transduced with shCtl[1], shCtl[2] or shADAM10. AB12 cells are stimulated with ionomycin (5 µM) or vehicle control. Actin was used as loading control of cell pellet fractions. **b**–**c** Representative gel lanes of western blot analysis of N-cad/FL in cell pellets and NTF in CM of AB12 (**b**) or PM27 (**c**) cells treated or not with GI254023X (5 µM), with ionomycin or vehicle control. Actin was used as loading control of cell pellet fractions. **d** Measurement of AB12 cell migration by transwell chamber assay challenged by CM of shCtl or shADAM10-transduced AB12 cells, in presence or not of recombinant mouse N-cadherin (rN-cadh; 500 ng/ml) (*n* = 3). Quantification of cell numbers per field is expressed as percentage of control condition (mean ± SEM); one-way ANOVA test; **P < 0.01; *** *P* < 0.001. **e** Transwell chamber assay in response to CM of shCtl AB12 cells treated or not with PD173074 (5 µM), in presence or not of recombinant mouse N-cadherin (rN-cadh; 500 ng/ml) (*n* = 3). Quantification of numbers of cells per field is expressed as percentage of control condition (mean ± SEM); one-way ANOVA test; ***P < 0.001

As N-cadherin is a substrate of ADAM10 and given its putative role in cell migration [21], we investigated a potential relationship between ADAM10 and N-cadherin in our model of mesothelioma cell migration. ShADAM10 and shCtl AB12 cells were therefore stimulated to produce the soluble 95kDa N-terminal N-cadherin fragment (NTF) with ionomycin (a calcium ionophore known to stimulate ADAM10 activity) [24, 25]. As ionomycin induced stimulation of several low molecular weight cytokines, conditioned media (CM) were concentrated by using 50 kDa filters, allowing a selection of molecules with a MW > 50 kDa. Interestingly, NTF fragment of N-cadherin was detected in CM when AB12 cells were stimulated with ionomycin as compared to cells stimulated with vehicle alone (Fig. 6a). However, the production of NTF fragment was markedly reduced in ADAM10-downregulated cells (Fig. 6a). Treatment of AB12 and PM27 cells with GI254023X also reduced NTF secretion (Fig. 6b, c). These results highlight that ADAM10 sheddase activity on N-cadherin is needed to generate soluble NTF in murine mesothelioma cells. In order to assess the effects of soluble NTF on mesothelioma cell migration, CM of ionomycin-stimulated shCtl or shADAM10 AB12 cells were used. Stimulation of AB12 cells with CM from shCtl cells induced a significant increase of AB12 cell migration (Fig. 6d). In sharp contrast, CM from shADAM10 cells was not able to promote AB12 cell migration (Fig. 6d). Interestingly, the addition of recombinant N-terminal fragment of N-cadherin to CM from shADAM10 cells restored the enhanced AB12 cell migration (Fig. 6d). The soluble fragment of N-cadherin was shown to be able to interact and stabilize FGFR (*Fibroblast Growth Factor Receptor*) to promote migration of cancer cells [26]. Therefore, the effect of a specific FGFR inhibitor, PD173074, on cell migration was investigated and showed that the pro-migratory effect of CM from shCtl AB12 cells was abolished by this inhibitor (Fig. 6e). Moreover, the addition of recombinant N-terminal fragment of N-cadherin was not able to counteract the inhibition of mesothelioma cell migration induced by FGFR inhibitor (Fig. 6e).

### ADAM10 is overexpressed in human mesothelioma cell line and ADAM10 depletion by siRNA decreases migration of human mesothelioma cells but not non-malignant mesothelial cells

**Fig. 7:**
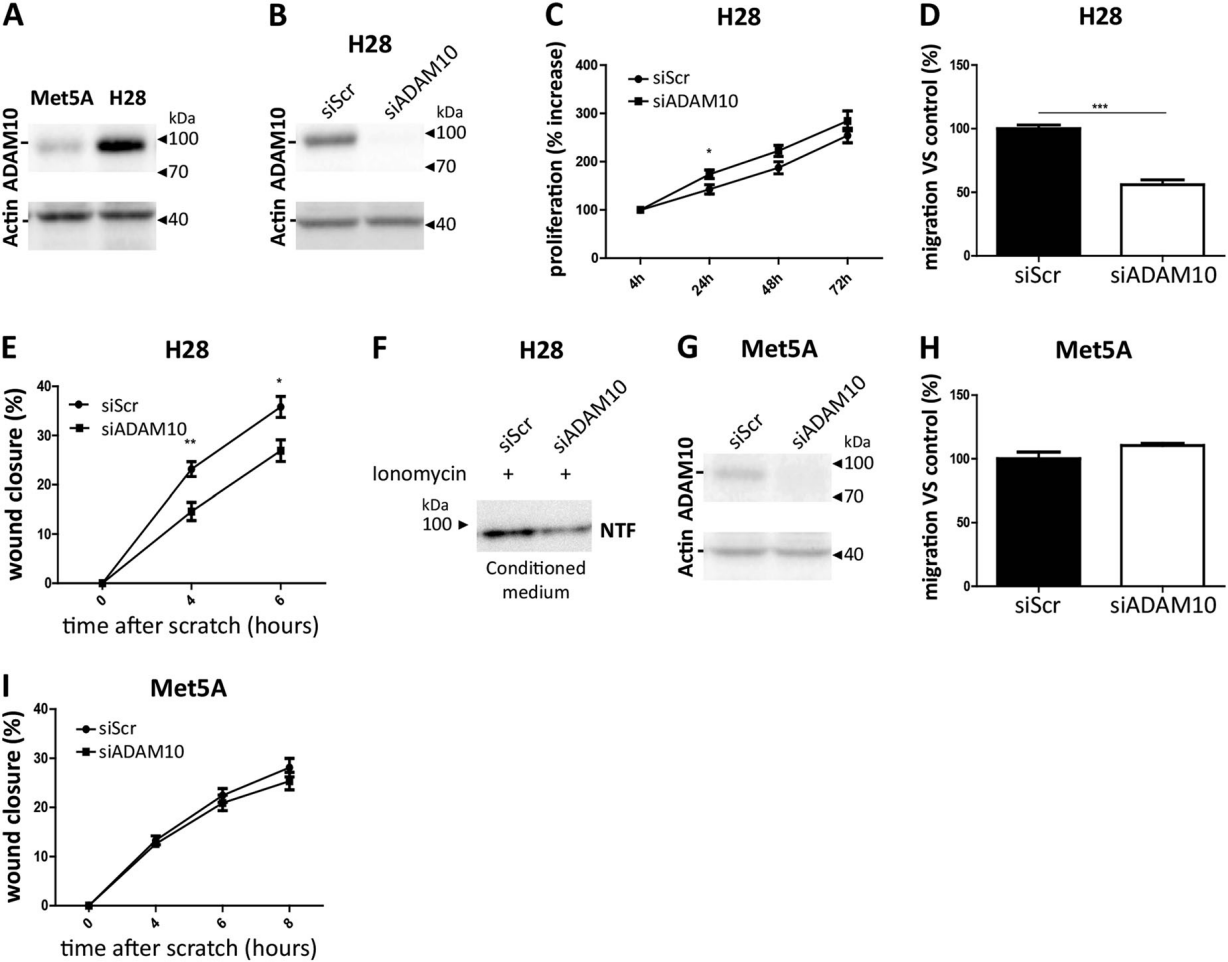
ADAM10 expression levels in human non-malignant mesothelial Met5A cells and human mesothelioma H28 cells and effects of siRNA targeting ADAM10 on migration of these cells. **a** Measurement of ADAM10 expression by western blot analysis in human Met5A and H28 cell lines. Actin was used as loading control. **b** Western blot analysis of ADAM10 protein levels in mesothelioma H28 cells transfected with siRNA control Scramble (siScr) or siRNA targeting ADAM10 (siADAM10). Actin was used as a loading control. **c** H28 proliferation rates were assessed 4, 24, 48 and 72 h after cell seeding by CYQUANT analysis (*n* = 6). All measurements were normalized to the ‘4 h-timing’ considered as baseline proliferation and expressed as percentage increase from baseline; Student *t* test; *P < 0.05. **d** Quantification of migrating H28 cell numbers per field (*n* = 3) by transwell assay. H28 cells were previously transfected with siScr or siADAM10. Results are expressed as percentage of control condition (mean ± SEM); Student *t* test; ***P < 0.001. **e** Wound healing assay performed on siScr or siADAM10-treated H28 cells. The wound closure was evaluated 4 and 6 h after the scratch (*n* = 9). Results are expressed as mean ± SEM; Student *t* test; *P < 0.05; **P < 0.01. **f** Representative gel lanes of western blot analysis of soluble N-terminal 95 kDa fragment (NTF) in conditioned medium of ionomycin-stimulated (3 µM) H28 cells transfected with siScr or siADAM10. **g** Western blot analysis of ADAM10 protein levels in mesothelial Met5A cells transfected with siRNA control Scramble (siScr) or siRNA targeting ADAM10 (siADAM10). Actin was used as a loading control. **h** Quantification of numbers of migrating Met5A cells transfected with siScr or siADAM10 per field by transwell assay (*n* = 3). Results are expressed as percentage of control condition (mean ± SEM). **i** Wound healing assay performed on siScr or siADAM10-treated Met5A cells. The wound closure was evaluated 4, 6 and 8 h after the scratch (*n* = 9). Results are expressed as mean ± SEM

To confirm the relevance of the results to human, human mesothelioma H28 cells and human non-malignant mesothelial Met5A cells were used. Interestingly, ADAM10 expression analysis revealed increased ADAM10 protein production in mesothelioma H28 cells as compared to non-malignant mesothelial Met5A cells (Fig. 7a). ADAM10 silencing in mesothelioma H28 cells using siRNA (Fig. 7b) did not modify proliferation rates as compared to siScramble-treated cells except a slight difference at 24 h (Fig. 7c). Interestingly, mesothelioma H28 cell migration measured in transwell chambers and in a scratch assay was significantly decreased in siADAM10-treated H28 cells as compared to siScramble-treated H28 cells (Fig. 7d, e) confirming the results obtained with mouse cells. Moreover, the production of NTF fragment is also reduced when ADAM10 was silenced in human H28 cells (Fig. 7f). In sharp contrast, in non-malignant mesothelial cells Met5A, ADAM10 depletion by siRNA targeting ADAM10 (Fig. 7g) did not modulate migratory behaviour neither in transwell nor in scratch assays as compared to siScramble-treated Met5A cells (Fig. 7h, i).

## Discussion

MPM is an aggressive cancer associated with a rapid invasion of pleural tissues [27]. Pathophysiology of MPM progression remains to be elucidated and the present study was designed to provide a contribution to a better understanding of disease mechanisms.

We provide, for the first time, evidence that ADAM10 plays a key role in mechanisms leading to MPM progression. The role of ADAM10 is suggested by an enhanced ADAM10 expression in human and mouse MPM samples. Moreover, we demonstrate that ADAM10 influences MPM progression by strongly enhancing tumour cell migration and invasion. The key implication of ADAM10 in MPM progression in vivo is supported by the reduced tumour growth observed after the specific ADAM10 downregulation. We also report that the pro-migratory effects of ADAM10 on MPM cells are mediated by its sheddase activity towards N-cadherin that generates a soluble fragment (NTF), this latter being in fine the trigger of cell migration probably through an activation of FGFR.

In this research project, fresh human samples of MPM and CPL were collected during thoracoscopic explorations. The age difference between groups of patients included in this study are explained by the origin of CPL that were collected from patients affected by persistent spontaneous pneumothorax requiring thoracoscopic explorations that are generally younger as compared to patients affected by MPM [28, 29]. For evident ethical reasons, it was indeed not possible to match the ages of the cohorts. The authors cannot exclude that part of the difference in ADAM10 expression levels in human samples might be due to aging since variations of ADAM10 expression have been reported in human platelets [30]. However, it appears that impact of aging on ADAM10 expression depends on cell type, since ADAM10 protein levels showed no age-associated regulation in human fibroblasts [31]. In the present study, ADAM10 overexpression in mesothelioma is supported by both in vitro and in vivo results, in particular in tissues derived from age-matched mice.

We could confirm the ADAM10 overexpression, found in human mesothelioma samples, when AB12 and PM27 mouse mesothelioma cells and H28 human mesothelioma cells were cultured in vitro but also when AB12 and PM27 were studied in vivo in an experimental mouse model where mesothelioma cells were intrapleurally implanted. This experimental model summarizes the main morphological and histological features of human MPM, and is appropriate to study mesothelioma pathology [32]. Biological functions of ADAM10 in MPM were investigated by inhibition studies using shRNA and an ADAM10 pharmacological inhibitor (GI254023X) in AB12 and PM27 mesothelioma cells as well as in H28 cells by using siADAM10 [6, 33].

This study is the first report supporting a potential role for ADAM10 in the pathophysiology of MPM progression. Although ADAM10 overexpression was reported in other malignancies [6–8, 10], the implication of ADAM10 in proliferation of cancer cells appears to be dependent on the cancer type. Indeed, Mullooly et al. [6] reported that ADAM10 downregulation induced no significant effect on breast cancer cell proliferation. In contrast, siRNA-targeted ADAM10 downregulation was reported to suppress cell proliferation of hepatocellular carcinoma [8], bladder cancer [7] and nasopharyngeal carcinoma [10] cells. In MPM, we showed that mesothelioma cell (AB12, PM27 and H28) proliferation is not modified when ADAM10 is targeted with shADAM10, GI254023X or siADAM10. Distribution of the different phases of the cell cycle was also not affected in mouse cells indicating that ADAM10 is not a major regulator of cell proliferation in mesothelioma progression.

Importantly, we could demonstrate using different experimental settings (transwell chambers, wound healing and spheroid assays), different mouse and human mesothelioma cells as well as different ADAM10 inhibition strategies (shADAM10, GI254023X or siADAM10) that ADAM10 enhances migration and invasion of mesothelioma cells. Supporting our findings, a role of ADAM10 in cell migration/invasion has been reported in several other type of cancers [6–8, 10, 15, 21].

In vivo results obtained during the present study largely contribute to demonstrate the key role of ADAM10 in MPM progression. Indeed, bioluminescence imaging and macroscopic analysis of tumours at the time of sacrifice revealed that tumours developed after intrapleural injection of shADAM10-treated AB12 cells are significantly smaller as compared to tumours developed after injection of shCtls-treated cells. These data present for the first time ADAM10 as a target of interest to counteract mesothelioma.

GI254023X, blocking ADAM10 catalytic activity, drastically reduced MPM cell migration and invasion indicating that ADAM10-related molecular mechanisms underlying the regulation of MPM cancer cell migration and invasion are dependent on the metalloprotease domain activity. By inhibition experiments, we could further prove that ADAM10 activity contributes to release the N-cadherin soluble ectodomain in extracellular media that is responsible for the pro-migratory effects observed in our models. In line with previous data, the extracellular domain of N-cadherin has been described to promote cell motility of squamous epithelial cells [20] and to be a stimulating agent of endothelial [17] and glioblastoma [21] cell migration.

In this study, we report, using a FGFR specific inhibitor (PD173074) [34], that FGF receptor is, via the soluble N-cadherin fragment, a key mediator for mesothelioma cell migration. The implication of FGF-dependent pathways in progression of MPM is consistent with previous data showing that the N-cadherin fragment can stabilize FGF receptors leading to a stimulation of migration in several cancer cells [26, 35].

This signalling cascade induced by ADAM10 protease could also be targeted in mesothelioma as development of selective FGFR inhibitors for clinical trials is in progress [36, 37]. The biology of FGFR pathways is complex and response to inhibitors depends on several factors and cancer type. Interestingly, in mesothelioma cell lines, it could be possible to determine a subgroup of MPM cells sensitive to FGFR inhibition [38].

Altogether, the results generated in this study show that ADAM10 protease activity is a key factor in the pathophysiological mechanisms leading to MPM progression and support the involvement of an ADAM10-generated pro-migratory soluble N-cadherin fragment acting via the FGFR pathway. In a therapeutic perspective, other studies are needed to assess whether this new ADAM10-driven pathophysiological mechanism in MPM could be targeted specifically by the use of ADAM10 inhibitors [39–41].

## Materials and methods

### Human samples

The study protocol was approved by the Ethical Committee of the University Hospital of Liège. Human pleural biopsies were collected during thoracoscopic explorations performed for diagnosis and therapy of pleural tumours, pleural effusions or persistent spontaneous pneumothorax. Samples obtained from patients explored for spontaneous pneumothorax were considered as CPL. Frozen pleural samples were used for RNA and protein extractions: 6 samples for CPL (5 for protein samples due to a lack of proteins for one patient) and 13 for MPM. Paraffin-embedded samples were used for immunohistochemistry analysis and are 4 for CPL and 18 for MPM (Table 1). The three histological types of MPM (epithelioid, biphasic and sarcomatoid) are represented in MPM samples used (Table 1). The proportion of these histological types is representative of epidemiologic data [3]. Patient’s characteristics are shown in Table 1.

### Cell lines and reagents

Murine mesothelioma AB12 cells (Sigma-Aldrich St. Louis, MO, USA) and human mesothelioma H28 cells (#CRL-5820, American Type Culture Collection (ATCC), Manassas, VA, USA) were cultured in RPMI 1640 medium (Lonza, Verviers, Belgium) supplemented with 1% L-Glutamine, 1% penicillin-streptomycin, 25 mM HEPES and 10% fetal bovine serum (FBS) (Gibco, Paisley, UK). Human non-cancerous mesothelial Met5A cells (#CRL-9444, ATCC) were cultured in Medium 199 containing 1.5 g/L sodium bicarbonate (Gibco) supplemented with 3.3 nM EGF, 400 nM hydrocortisone, 1% penicillin-streptomycin, 20 mM HEPES and 10% FBS. Primary Mesothelioma 27 (PM27) cells, generated as described below (see ‘Primary cancerization mouse model and isolation of primary mesothelioma cells’ section), were cultured in DMEM (Gibco) supplemented with 1% L-Glutamine, 1% penicillin-streptomycin and 10% FBS. Cell cultures were incubated at 37 °C in a 5% CO_2_ atmosphere and routinely tested for mycoplasma contamination. GI254023X (Sigma-Aldrich) [42], ionomycin (Sigma-Aldrich) and PD173074 (Abcam, Cambridge, UK) were prepared in DMSO (vehicle). For assays in presence of GI254023X or vehicle, mesothelioma cells were pretreated 16 h before the experiment. Recombinant mouse N-cadherin/Fc Chimera Protein was reconstituted in PBS (R&D Systems, Minneapolis, USA).

### Plasmid, lentiviral vector generation and transduction

Gene transfer lentiviral plasmid pSIN-SFFV-GFP Ub-EmGFP was kindly provided by Dr. Yasuhiro Ikeda (Mayo Clinic, Rochester, MN). This plasmid allows the dual Emerald GFP and firefly luciferase expression. Lentiviral transfer shRNA plasmids directed against mouse ADAM10 (TRCN0000031848, TRCN0000031844) and non-target control shRNA were purchased from Sigma-Aldrich (SHC005 and SHC012). Lentiviral vectors (rLV) were generated by the GIGA Viral Vectors platform (University of Liège, Belgium). AB12 cells were first transduced with rLV allowing the dual expression of EmGFP and luciferase. Transduced cells were selected by FACS using EmGFP signal and then transduced with rLV shRNA (TRCN0000031848, SHC005 and SHC012) and selected by puromycin (Invivogen, ant-pr-1). PM27 cells were transduced with shRNA rLV (TRCN0000031844, SHC005 and SHC012) and selected by puromycin.

### siRNA transfections

Human cells were transiently transfected with the SMARTpool ON-TARGETplus ADAM10 siRNA (Dharmacon) or the corresponding scrambled control siRNA (Dharmacon). A volume of 20 nM of siRNA were transfected into cells with the help of Lipofectamine RNAiMAX Transfection Reagent (ThermoFisher Scientific, Carlsbad, CA, USA) according to manufacturer’s instructions.

### Mouse models

Protocols used in this study were approved by the Animal Ethical Committee of the University of Liège and male BALB/cJRj mice aged 6 to 8 weeks (Janvier Labs, St. Berthevin, France) have been used. Mice were randomly divided into experimental groups. Total sample size was determined with the ‘G Power’ software using the “Wilcoxon–Mann–Whitney” type *t* test.

### Primary cancerization mouse model and isolation of primary mesothelioma cells

Primary mesothelioma were obtained from BALB/cJRj mice subjected to repeated peritoneal injections of UICC crocidolite asbestos fibres (SPI Supplies, West Chester, PA, USA) (adapted from work of Testa group [43, 44]). Briefly, mice were subjected to eight intraperitoneal injections, every three weeks, of 400 µg of fibres in 300 µl PBS. Crocidolite-exposed mice were sacrificed maximum one year after the first injection and ascites have been collected for primary mesothelioma cell culture.

### Pleural mesothelioma mouse model

Mice were intrapleurally injected with mesothelioma AB12 or PM27 cells. A surgical incision was made into the right chest wall followed by an injection of cells in the pleural cavity through an intercostal space (1.5 × 10^6^ AB12 cells or 10^6^ PM27 cells in 200 µl serum-free medium). Control mice were injected with serum-free medium only and diaphragm, which is lined by a mesothelium, was used as CPL because dissecting only the mesothelial cell monolayer in mice is technically not possible. Mice were sacrificed 40 or 21 days after AB12 or PM27 tumour cell inoculation, respectively.

### Isolation of mesothelial cells

Normal mouse mesothelial cells have been isolated from BALB/cJRj mouse peritoneal cavity as previously described [45].

### In vivo BioLuminescence optical Imaging (BLI)

Bioluminescence was detected in animals injected with AB12 cells expressing luciferase gene as described previously [46] with the in vivo Imaging System IVIS 200 (Xenogen Corp.; Alameda, CA, USA). The extent of tumour development was assessed by determining regions of interest (ROI) and quantifying bioluminescent intensity using Living Image Software (Caliper Life Sciences, Inc.) Results are expressed in photon counts per area (photons/s/cm2/sr).

### Protein and RNA extractions from mouse and human samples

Tissue homogenisation of mesothelioma tumour samples was carried out using a Mikro-Dismembrator (B. Braun, Biotech International, Germany). Protein extractions were performed with 8 M urea buffer containing a protease inhibitor cocktail (cOmplete^TM^, Roche, Mannheim, Germany). To extract proteins from cultured cells, RIPA buffer (50 mM Tris, 150 mM NaCl, 1% NP40, 1% Triton X100, 1% sodium deoxycholate, 0,1% SDS) containing a protease inhibitor cocktail (cOmplete^TM^) was used. RNA extractions were performed using Trizol Reagent (Ambion; Carlsbad, CA, USA) and extracted products were purified on columns according to manufacturer’s instructions (RNeasy Mini Kit; Qiagen, Hilden, Germany).

### RT-PCR

Primers used to detect mouse and human ADAM10 transcripts were 5′TTT-GGA-TCC-CCA-CAT-GAT-TCT-G3′ and 5′GGT-TGG-CCA-GAT-TCA-ACA-AAA-C3′. The primers were designed as previously described [47]. Reverse transcriptase polymerase chain reaction was done using Tth DNA Polymerase kit (Roche). Quantification of each band density was performed using Quantity-One software (Bio Rad Laboratories, Hercule, CA, USA). ADAM10 mRNA levels were normalized to the 28S rRNA band, used as internal standard.

### Immunohistochemistry

Human fresh tissues were fixed in a 4% paraformaldehyde solution. Tissue sections (5 µm) were treated with target retrieval buffer (Dako, Carpinteria, CA, USA) followed by endogenous peroxydase activity blockade with 3% hydrogen peroxide (Merck, Darmstadt, Germany). Non-specific binding sites were blocked with universal blocker (BioGenex, Fremont, CA, USA). Rabbit anti-ADAM10 antibody (ab1997, Abcam, Cambridge, UK, 1/1000) was incubated 2 h at room temperature (RT) followed by incubation with a swine anti-rabbit horseradish peroxidase (HRP)-linked antibody (Dako, 1/3000) for 30 min at RT. Sections were then stained with 3,3′ diaminobenzidine tetrachloride (DAB) (Dako) and digitalized using NanoZoomer 2.0-HT system (0.23 µm/pixel (×40) scanning resolution). Quantification of ADAM10 staining in each sample was based on a score of staining intensity ranging from 0 (absence of staining) to 3 (strong staining).

### Immunocytochemistry

PM27 cells (2 × 10^4^cells/100 µl) were cultured on microscopy slides during 24 h, fixed, permeabilized and incubated for 1 h with following primary antibodies: rabbit anti-mesothelin (250519, Abbiotec; 1/200); rabbit anti-keratin5 (PRB-160P, Covance; 1/100); guinea pig anti-vimentin (2220900801, Quartett; 1/250). Secondary goat anti-rabbit and rabbit anti-guinea pig antibodies were added. Sections were then stained with DAB and digitalized using NanoZoomer 2.0-HT system (0.23 µm/pixel (40 × ) scanning resolution).

### Western blot

After electrophoresis on 12% acrylamide gel, proteins were transferred on nitrocellulose PVDF membrane (Perkin Elmer Life Sciences, Waltham, MA, USA). Primary antibodies used were rabbit anti-ADAM10 (Abcam; 1/1000) or rabbit anti-N-cadherin (4061, Cell signalling; 1/500). Revelation was performed by chemiluminescence after incubation with HRP-conjugated swine anti-rabbit secondary antibody (Dako, 1/3000). Actin or GAPDH were detected as a loading control.

### Proliferation assay

DNA content was measured using CYQUANT Cell Proliferation Assay kit (Thermo Fisher Scientific) according to manufacturer’s instructions. Cell proliferation was evaluated 4, 24, 48 and 72 h after cell seeding in 96-well plates (2000 cells/well). Plates were read with microplate Gemini EM spectrofluorometer (Molecular Devices, USA).

### Cell cycle analysis

Cells (3 × 10^5^) were cultured in 6-well plates during 24 h, in presence or not of GI254023X (5 µM) or vehicle. Cells were then harvested and fixed in 70% ethanol at −20 °C overnight. Samples were treated with 50 µg/ml RNase during 30 min at 37 °C and stained with 20 µg/ml propidium iodide (PI, Sigma-Aldrich) followed by flow cytometry on FACS Calibur (BD Biosciences). The percentage of cells in each cell cycle phase (G1, S or G2) was calculated using the Modfit software (Verity Software House, Topsham, ME, USA) [48].

### N-cadherin ectodomain shedding assay

10^6^ mesothelioma AB12, PM27 or H28 cells were seeded in 100 mm culture dishes. Forty-eight hours after seeding, the cells were washed twice with PBS and serum-free medium was added. The cells were treated with ionomycin (5 µM or 3 µM for H28 cells) or vehicle during 2 h, in presence or not of GI254023X (5 µM). Media conditioned by those cells (CM) were harvested and proteins of cell pellets were extracted. These CM were concentrated by using Amicon Ultra Centrifugal Filter Units 50 kDa according to manufacturer’s instructions (Merck, Ireland).

### Transwell chamber assay

Cell suspensions (AB12, PM27, H28 and Met5A) were seeded in serum-free media (10^5^ cells/200 µl) in the upper compartment of inserts (8 µm pore size, Costar; Kennebunk, ME, USA). The lower chambers of the transwell were filled either with medium supplemented by 10% FBS used as a chemoattractant or with RPMI with 1% FBS and 1/25 concentrated AB12 CM complemented or not with 500 ng/ml of recombinant mouse N-cadherin/Fc Chimera Protein. In some assays, GI254023X (5 µM), PD173074 (5 µM) or vehicles were added. Chambers were incubated at 37 °C for 6 h or 16 h (when CM were filled in the lower chambers). Migrating cells were fixed, stained with Giemsa and digitalized using NanoZoomer 2.0-HT system (0.23 µm/pixel (40 × ) scanning resolution). Migrating cells were counted on eight random fields of each membrane at 20 × (3 membranes/condition) and results are expressed as percentage of control condition.

### Scratch assay

5 × 10^5^ mesothelioma or Met5A cells/well were seeded in 6-well plates in complete media. When cells reached confluency, scratches were made using pipette tips. Cells were rinsed and incubated in medium containing 2% FBS, 10^–6^M cytosine β-D arabinofuranoside hydrochloride, an antineoplastic agent, and, in some cases, GI254023X (5 µM) or vehicle. The closure of the wound was monitored 0, 4, 6 and 8 h after the scratch (2 random pictures were taken per wound, at least 4 wounds/condition) using an inverted microscope (Eclipse TI-S; Nikon, Tokyo, Japan). Area of the scratch was quantified at 0, 4, 6 and 8 h using NIS-Elements BR 3.2 Software and results were expressed as percentage of wound closure of mean value of at least eight independent measurements.

### Spheroid assay

Multicellular spheroids were obtained from AB12 or PM27 cells (1.5 × 10^3^ cells) embedded in non-adherent 96-well plates in media containing carboxymethylcellulose (Sigma-Aldrich) as previously described [49]. Spheroids were then embedded in type I collagen gels (Corning; Bedford, MA, USA) treated, for some experiments, with GI254023X (5 µM) or vehicle. Twenty-four hours after culture, photographs were taken using an inverted microscope (Eclipse TI-S; Nikon, Tokyo, Japan). The data are analysed as described in Carnet et al., 2015 [49].

### Statistical analysis

Gaussian distribution was tested with Kolmogorov–Smirnov test. The statistical analysis were performed using two-tailed unpaired Student *t* test or ANOVA test with GraphPad Prism Software (La Jolla, CA, USA) and *p* < 0.05 is considered as significant. The results are expressed as mean ± SEM and are representative of at least two or three experiments performed independently.

## Data Availability

All protocols and material generated in this study is available in our registries (Laboratory notebooks #30500, #23847, #16966, #382019, #38030, #60245).

## References

[1] Bononi A, Napolitano A, Pass HI, Yang H, Carbone M (2015). Latest developments in our understanding of the pathogenesis of mesothelioma and the design of targeted therapies. Expert Rev Respir Med.

[2] Donaldson K, Poland CA, Murphy FA, Macfarlane M, Chernova T, Schinwald A (2013). Pulmonary toxicity of carbon nanotubes and asbestos—similarities and differences. Adv Drug Deliv Rev.

[3] Ray M, Kindler HL (2009). Malignant pleural mesothelioma: an update on biomarkers and treatment. Chest.

[4] Yap TA, Aerts JG, Popat S, Fennell DA (2017). Novel insights into mesothelioma biology and implications for therapy. Nat Rev Cancer.

[5] Andreini C, Banci L, Bertini I, Elmi S, Rosato A (2005). Comparative Analysis of the ADAM and ADAMTS Families. J Proteome Res.

[6] Mullooly M, Mcgowan PM, Kennedy SA, Madden SF, Crown J, Donovan NO (2015). ADAM10: a new player in breast cancer progression?. Br J Cancer.

[7] Fu L, Liu N, Han Y, Xie C (2014). ADAM10 regulates proliferation, invasion, and chemoresistance of bladder cancer cells. Tumor Biol.

[8] Liu S, Zhang WEI, Liu KAI, Ji BAI, Wang G (2015). Silencing ADAM10 inhibits the in vitro and in vivo growth of hepatocellular carcinoma cancer cells. Mol Med Rep.

[9] Guo J, He L, Yuan P, Wang P, Lu Y, Tong F (2012). ADAM10 overexpression in human non-small cell lung cancer correlates with cell migration and invasion through the activation of the Notch1 signaling pathway. Oncol Rep.

[10] You B, Shan Y, Shi S, Li X, You Y (2015). Effects of ADAM10 upregulation on progression, migration, and prognosis of nasopharyngeal carcinoma. Cancer Sci.

[11] Dreymueller D, Uhlig S, Ludwig A (2015). DAM-family metalloproteinases in lung inflammation: potential therapeutic targets. Am J Physiol Lung Cell Mol Physiol.

[12] Pruessmeyer J, Hess FM, Alert H, Groth E, Pasqualon T, Schwarz N (2014). Leukoc require ADAM10 but Not ADAM17 their Migr Inflamm Recruit into alveolar Space.

[13] Rocks N, Paulissen G, El Hour M, Quesada F, Crahay C, Gueders M (2008). Emerging roles of ADAM and ADAMTS metalloproteinases in cancer. Biochim.

[14] Paulissen G, Rocks N, Gueders MM, Crahay C, Quesada-Calvo F, Bekaert S (2009). Role of ADAM and ADAMTS metalloproteinases in airway diseases. Respir Res.

[15] Moss ML, Stoeck A, Yan W, Dempsey PJ (2008). ADAM10 as a target for anti-cancer therapy. Curr Pharm Biotechnol.

[16] Yilmaz M, Christofori G (2010). Mechanisms of motility in metastasizing cells. Mol Cancer Res.

[17] Derycke L, Morbidelli L, Ziche M, De Wever O, Bracke M, Van Aken E (2006). Soluble N-cadherin fragment promotes angiogenesis. Clin Exp Metastas-.

[18] Paradies NE, Grunwald GB (1993). Purification and characterization of NCAD90, a soluble endogenous form of N-cadherin, which is generated by proteolysis during retinal development and retains adhesive and neurite-promoting function. J Neurosci Res.

[19] Utton MA, Eickholt B, Howell FV, Wallis J, Doherty P (2001). Soluble N-cadherin stimulates fibroblast growth factor receptor dependent neurite outgrowth and N-cadherin and the fibroblast growth factor receptor co-cluster in cells. J Neurochem.

[20] Kim J, Islam S, Kim YJ, Prudoff RS, Sass KM, Wheelock MJ (2000). N-Cadherin extracellular repeat 4 mediates epithelial to mesenchymal transition and increased motility. J Cell Biol.

[21] Kohutek ZA, Charles G, Redpath GT, Hussaini IM (2009). ADAM-10-ediated N-Cadherin cleavage is protein kinase C- ␣ dependent and promotes glioblastoma cell migration. J Neurosci.

[22] Yaziji H, Battifora H, Barry TS, Hwang HC, Bacchi CE, Mcintosh MW (2006). Evaluation of 12 antibodies for distinguishing epithelioid mesothelioma from adenocarcinoma: identification of a three-antibody immunohistochemical panel with maximal sensitivity and specificity. Mod Pathol.

[23] Shackleton B, Crawford F, Bachmeier C (2016). Inhibition of ADAM10 promotes the clearance of Aβ across the BBB by reducing LRP1 ectodomain shedding. Fluids Barriers CNS.

[24] Marambaud P, Shioi J, Serban G, Georgakopoulos A, Sarner S, Nagy V (2002). A presenilin-1/g-secretase cleavage releases the E-cadherin intracellular domain and regulates disassembly of adherens junctions. EMBO J.

[25] Reiss K, Maretzky T, Ludwig A, Tousseyn T, Strooper B De, Hartmann D (2005). ADAM10 cleavage of N-cadherin and regulation of cell-cell adhesion and b-catenin nuclear signalling. EMBO J.

[26] Suyama K, Shapiro I, Guttman M, Hazan RB (2002). A signaling pathway leading to metastasis is controlled by N-cadherin and the FGF receptor. Cancer Cell.

[27] Zhang W, Wu X, Wu L, Zhang W, Zhao X (2015). Advances in the diagnosis, treatment and prognosis of malignant pleural mesothelioma. Ann Transl Med.

[28] Huang YH, Chang PY, Wong KS, Chang CJ, Lai JY, Chen JC (2017). An age-stratified longitudinal study of primary spontaneous pneumothorax. J Adolesc Health.

[29] Schnell J, Koryllos A, Lopez-Pastorini A, Lefering R, Stoelben E (2017). Spontaneous pneumothorax. Dtsch Arztebl Int.

[30] Schuck F, Wolf D, Fellgiebel A, Endres K (2016). Increase of alpha-Secretase ADAM10 in platelets along cognitively healthy aging. J Alzheimers Dis.

[31] Kern A, Roempp B, Prager K, Walter J, Behl C (2006). Downregulation of endogenous amyloid precursor protein processing due to cellular aging. J Biol Chem.

[32] Mezzapelle R, Rrapaj E, Gatti E, Ceriotti C, Marchis FDe, Preti A (2016). Human malignant mesothelioma is recapitulated in immunocompetent BALB/c mice injected with murine AB cells. Sci Rep.

[33] Hundhausen C, Misztela D, Berkhout TA, Broadway N, Saftig P, Reiss K (2003). The disintegrin-like metalloproteinase ADAM10 is involved in constitutive cleavage of CX3CL1 (fractalkine) and regulates CX3CL1-mediated cell-cell adhesion. Blood.

[34] Sun Y, Fan X, Zhang Q, Shi X, Xu G, Zou C (2017). Cancer-associated fibroblasts secrete FGF-1 to promote ovarian proliferation, migration, and invasion through the activation of FGF-1/FGFR4 signaling. Tumor Biol..

[35] Nguyen T, Mege RM (2016). N-Cadherin and fibroblast growth factor receptors crosstalk in the control of developmental and cancer cell migrations. Eur J Cell Biol.

[36] Chae YK, Ranganath K, Hammerman PS, Mohindra N, Kalyan A, Matsangou M (2017). Inhibition of the fibroblast growth factor receptor (FGFR) pathway: the current landscape and barriers to clinical application. Oncotarget.

[37] Brooks AN, Kilgour E, Smith PD (2012). Molecular pathways: fibroblast growth factor signaling: a new therapeutic opportunity in cancer. Clin Cancer Res.

[38] Quispel-Janssen JM, Badhai J, Schunselaar L, Price S, Brammeld J, Iorio F (2018). Comprehensive pharmacogenomic profiling of malignant pleural mesothelioma identifies a subgroup sensitive to FGFR inhibition.. Clin Cancer Res.

[39] Moss ML, Bomar M, Liu Q, Sage H, Dempsey P, Lenhart PM (2007). The ADAM10 prodomain is a specific inhibitor of ADAM10 proteolytic activity and inhibits cellular shedding events. J Biol Chem.

[40] Madoux F, Dreymuller D, Pettiloud J, Santos R, Ludwig A, Fields GB (2016). Discovery of an enzyme and substrate selective inhibitor of ADAM10 using an exosite-binding glycosylated substrate. Sci Rep.

[41] Dreymueller D, Ludwig A (2017). Considerations on inhibition approaches for proinflammatory functions of ADAM proteases. Platelets.

[42] Vincent B (2016). Regulation of the α-secretase ADAM10 at transcriptional, translational and post-translational levels. Brain Res Bull.

[43] Altomare DA, You H, Xiao G, Ramos-nino ME, Skele KL, Rienzo ADe (2005). Human and mouse mesotheliomas exhibit elevated AKT/PKB activity, which can be targeted pharmacologically to inhibit tumor cell growth. Oncogene.

[44] Altomare DA, Vaslet CA, Skele KL, Rienzo ADe, Devarajan K, Jhanwar SC (2005). Priority report a mouse model recapitulating molecular features of human mesothelioma. Cancer Res.

[45] Bot J, Whitaker D, Vivian J, Lake R, Yao V, Mccauley R (2003). Animal and in vitro models in human diseases culturing mouse peritoneal mesothelial cells. Pathol Res Pr.

[46] Donati K, Sépult C, Rocks N, Blacher S, Gérard C, Noel A (2017). Neutrophil-derived interleukin 16 in premetastatic lungs promotes breast tumor cell seeding. Cancer Growth Metastas-.

[47] Rocks N, Paulissen G, Quesada Calvo F, Polette M, Gueders M, Munaut C (2006). Expression of a disintegrin and metalloprotease (ADAM and ADAMTS) enzymes in human non-small-cell lung carcinomas (NSCLC). Br J Cancer.

[48] Otjacques E, Binsfeld M, Rocks N, Blacher S, Vanderkerken K, Noel A (2013). Mithramycin exerts an anti-myeloma effect and displays anti-angiogenic effects through up-regulation of anti-angiogenic factors. PLoS ONE.

[49] Carnet O, Lecomte J, Masset A, Primac I, Durré T, Maertens L (2015). Mesenchymal stem cells shed amphiregulin at the surface of lung carcinoma cells in a juxtacrine. NEO.

